# Incident tuberculosis disease in patients receiving biologic therapies in the Western Cape, South Africa from 2007 to 2018

**DOI:** 10.1186/s12879-020-05624-0

**Published:** 2020-11-30

**Authors:** Tessa du Toit, Tonya M. Esterhuizen, Nicki Tiffin, Ahmed A. Abulfathi, Helmuth Reuter, Eric H. Decloedt

**Affiliations:** 1 Division of Clinical Pharmacology, Department of Medicine, Faculty of Medicine and Health Sciences, Stellenbosch University, PO Box 241, Cape Town, 8000 Republic of South Africa; 2 Division of Epidemiology and Biostatistics, Faculty of Medicine and Health Sciences, Stellenbosch University, Stellenbosch, South Africa; 3 Computational Biology Division, Integrative Biomedical Sciences, University of Cape Town, Cape Town, South Africa; 4 Wellcome Centre for Infectious Disease Research in Africa, Institute of Infectious Diseases and Molecular Medicine, University of Cape Town, Cape Town, South Africa; 5 Centre for Infectious Disease Epidemiology Research, School of Public Health and Family Medicine, University of Cape Town, Cape Town, South Africa; 6 Department of Clinical Pharmacology and Therapeutics, Faculty of Basic Clinical Sciences, College of Medical Sciences, University of Maiduguri, Maiduguri, Nigeria

**Keywords:** Tumour necrosis factor-alpha (TNF-α), Biologics, Tuberculosis, South Africa

## Abstract

**Background:**

South Africa has one of the highest tuberculosis incidence rates. Biologic disease-modifying anti-rheumatic drugs are associated with an increased risk of tuberculosis. The objective of this study was to describe the tuberculosis disease incidence rate among public sector patients receiving biologic therapies in the Western Cape Province.

**Methods:**

A retrospective, descriptive analysis was undertaken using routine health data collated by the Provincial Health Data Centre from January 2007 (first use of biologic therapy in the Western Cape) to September 2018.

**Results:**

We identified 609 patients treated with tumour necrosis factor-alpha (TNF-α) or non-TNF-α biologic therapies. Thirty-seven (37) patients developed tuberculosis after biologic therapy exposure, of whom the majority (78%) had an immune mediated inflammatory disease and the remainder (22%) a haematologic malignancy. The incidence rate of tuberculosis per 100,000 person-years was 2227 overall [95% confidence interval (CI): 1591, 3037]. Patients treated with TNF-α inhibitors and non-TNF-α inhibitors had estimated incidence rates of 2819 [95% CI: 1669, 4480] and 1825 [95% CI: 1131, 2797], respectively (*p* = 0.10).

**Conclusion:**

Patients exposed to both TNF-α and non-TNF-α biologic therapies may have a higher incidence of tuberculosis disease compared to the background risk of 681 cases per 100,000 per year in the Western Cape.

## Background

The efficacy of biologic therapies to treat immune-mediated inflammatory conditions [1–3] and certain haematologic malignancies [4, 5] has been established. Yet the use of biologic therapy is limited by the risk of opportunistic infections, in particular tuberculosis (TB) disease [1, 6–13], because it suppresses the immune response of the patient to pathogens. This is particularly relevant given the high background TB incidence in South Africa, which the World Health Organisation (WHO) ranks as one of the top TB burden countries globally with an estimated TB incidence rate of 567 per 100,000 population in 2017 [14].

For these reasons, guidelines recommend screening and prophylactic therapy for TB prior to initiation of biologic therapy [8, 15–17]. Biologic therapies specifically targeting tumour necrosis factor-alpha (TNF-α) are a recognised, independent risk factor for TB disease due to the central role of TNF-α in granuloma formation and maintenance [6, 10]. Anti-TNF-α monoclonal antibodies, particularly infliximab and adalimumab, have consistently been associated with the highest risk of developing TB disease [18–23]. Additionally, patients receiving biologic therapies may have an additional risk for developing TB due to their underlying disease (inflammatory condition or haematological malignancy) or its treatment including synthetic disease modifying anti-rheumatic drugs (DMARDs) and corticosteroid exposure [13, 18].

Current biologic therapy risk estimates for TB have mostly been assessed in low and intermediate TB burden countries [8, 20], but not in a high TB burden environment such as South Africa. The South African Biologics Registry (SABIO) captures biologic use data for South African patients receiving private health care, but these are generally individuals with higher socio-economic circumstances and lower risk of exposure to TB, and are not representative of the general South African patient population. When comparing the TB incidence rates in TNF-α inhibitors users in the SABIO registry, incidence was 1387 per 100,000 person-years, which is approximately 10-fold that of the British (BSRBR), French (RATIO) and Spanish (BIOBADASER) registries where the TB incidence ranges from 106 to 172 per 100,000 person-years [22, 24].

The Western Cape Province in South Africa has a particularly high TB burden, with an estimated incidence rate of 681 per 100,000 population in 2015 [25]. This study describes the incidence rate and time to TB onset among public sector patients receiving biologic therapies in this high-TB burden setting in the Western Cape.

## Methods

We conducted a retrospective, descriptive study of anonymised quantitative data from patients who received biologic therapies in the public health sector in the Western Cape, South Africa, as identified by the Provincial Health Data Centre (PHDC), a health information exchange that collates routine health data from a variety of Provincial health data platforms. The clinical data were de-identified and underwent data perturbation by the Provincial Health Data Centre prior to release to ensure that patients could not be re-identified from their health records. All patients receiving biologic therapies in the period from the first recorded biologic use in January 2007 to 30 September 2018, were included in the study irrespective of age, therapy type or indication. The biologic therapies included the TNF-α inhibitors infliximab, adalimumab, golimumab and etanercept, and the non-TNF-α inhibitor biologics rituximab, abatacept, tocilizumab and ustekinumab.

We analysed the following variables: baseline characteristics (age, gender, HIV status, dispensed isoniazid preventative therapy (IPT) and use of traditional DMARDS), biologic exposure (therapy type, treatment duration and any biologic swops) and outcome variables (TB disease diagnosis).

In South Africa an established IPT protocol exists for HIV positive patients only [26]. A 12-month duration of IPT is recommended in all non-pregnant HIV positive patients who screened negative for active TB based on symptoms, irrespective of their latent TB status. There is no formal IPT protocol for patients with rheumatologic or haematologic conditions.

The indications for biologic therapy were broadly grouped into immune-mediated inflammatory diseases (including rheumatoid arthritis, spondyloarthropathies and juvenile idiopathic arthritis) and haematologic malignancy, (including Non-Hodgkin Lymphoma and chronic lymphoid leukaemia) based on the combination of traditional DMARD or chemotherapeutic use. A rheumatologist (HR) led the classification process where dispensing records of traditional DMARDs (methotrexate, sulfasalazine and/ or chloroquine) in combination with any of the eight biologic therapies was classified as an immune mediated inflammatory disease (IMID). On the other hand, dispensing record of chemotherapeutic medications (cyclophosphamide, doxorubicin and/ or vincristine) in combination with rituximab use was classified as a haematologic malignancy.

Ascertainment of TB was based on digital health records compiled by the PHDC using the data from the National Health Laboratory Services, pharmacy dispensing records from the Western Cape Government Health electronic platform, and data from the electronic TB register (ETR).

Statistical analysis was undertaken using SPSS version 25.0 [27]. Categorical and numerical baseline variables were compared between therapy (TNF-α and non-TNF-α inhibitors) and indication groups (IMID and haematologic malignancy) using Chi-Square and Mann-Whitney U test respectively. Normality of distribution was tested using both Kolmogorov-Smirnov and Shapiro-Wilk tests. Incidence rates of TB were expressed as events per 100,000 person-years using OpenEpi online statistical calculator with Mid-P exact 95% confidence intervals (CI) [28]. The total, two indication groups (immune mediated inflammatory diseases and haematologic malignancy), two major therapy groups (TNF-α and non-TNF-α inhibitors) and individual biologic therapy incidence rates were calculated. Some patients were on multiple biologic therapies, therefore proportions and individual biologic therapy TB incidence rates were calculated based on the most recent biologic therapy used before or at the time of TB diagnosis. The median time to TB disease (expressed in months) from the start of the most recent biologic therapy used was estimated overall and for the individual biologic therapies. The relationship between time to developing TB and risk factors was modelled using Cox proportional hazards models. The data met proportional hazards assumptions. Independent association of predictor variables for TB incidence were estimated as adjusted hazard ratios and 95% CI.

## Results

During the study period, 611 patients received biologic therapies of which 609 patients were included in the study. One patient was excluded due to the date of death occurring prior to the date of ascertainment of TB, most likely indicating the lag time for TB culture from a sample taken just before death. A second patient was excluded based on a highly implausible combination of dispensed medications, which was likely an uncorrected dispensing record error.

The baseline variables, including age, gender, HIV status and isoniazid preventative therapy (IPT) are compared between indication and therapy groups as illustrated in Table 1**.** Of note, the significant differences in baseline characteristics between the two therapy groups were age, patients treated with TNF-α were younger than those treated with non-TNF-α inhibitors. Further, more males were treated with TNF-α and females with non-TNF-α inhibitors. Significantly more patients treated with TNF-α inhibitors received IPT compared to patients treated with non-TNF-α inhibitors. Regarding the indication groups, significant differences were also observed between age groups, where patients with an IMID were younger than patients with a haematologic malignancy. Significantly more patients with IMID received IPT than patients with haematologic malignancies. The outcome variables, TB and death, are also illustrated in Table 1. Significantly more patients who were treated with non-TNF-α inhibitors compared to TNF-α inhibitors, and who had a haematological malignancy compared to IMID, died in this study period.

**Table 1:** Baseline and outcome variables by therapy and indication groups

All patients *n* = 609 *n* (%)		Therapy groups *n* = 598^a^			Indication groups n = 609		
		TNF-α *n* = 174 n (%)	Non-TNF-α *n* = 424 n (%)	*p*^b^	IMID *n* = 348 n (%)	HM *n* = 261 n (%)	*p*
AGE IN YEARS at initiation of biologic therapy							
Median (IQR)	41 (22–57)	33 (18–46)	47 (25–59)	*< 0.001*	28 (13–45)	55 (41–63)	*< 0.001*
GENDER							
F	311 (51)	73 (42)	232 (55)	*0.005*	185 (53)	126 (48)	*0.223*
M	298 (49)	101 (58)	192 (45)		163 (47)	135 (52)	
HIV STATUS at initiation of therapy							
positive	45 (7)	13 (8)	31 (7)	*0.946*	27 (8)	18 (7)	*0.687*
negative	564 (93)	161 (92)	393 (93)		321 (92)	243 (93)	
IPT							
yes	211 (35)	119 (78)	83 (20)	*< 0.001*	206 (59)	5 (2)	*< 0.001*
no	398 (65)	55 (22)	341 (80)		142 (41)	256 (98)	
SURVIVAL at end of study period							
died	94 (15)	16 (9)	78 (18)	*0.005*	39 (11)	55 (21)	*0.001*
alive	515 (85)	158 (91)	346 (82)		309 (89)	206 (79)	
TB INCIDENCE after initiation of biologic therapy							
TB	37 (6)	16 (9)	19 (4)	*0.026*	29 (8)	8 (3)	*0.007*
no TB	572 (94)	158 (91)	405 (96)		319 (92)	253 (97)	

^a^patients receiving both TNF-α and non-TNF-α inhibitors (*n* = 11) were excluded

^b^*p* value compares TNF-α to non-TNF-α only (*n* = 598) using a Mann-Whitney test for age in years and Chi-Square tests for categorical variables

*TNF-α* tumour necrosis factor-alpha inhibitors, *non-TNF-α* non-tumour necrosis factor- alpha inhibitors, *IMID* immune mediated inflammatory disease, *HM* haematologic malignancy, *IPT* isoniazid preventative therapy

Of the 609 patients, 37 patients developed TB following initiation of biologic therapy. Four patients had two TB episodes, therefore a total of 41 TB episodes were observed in the study period. The total follow-up time to developing TB disease, death or study end date was 1662 person-years with a calculated TB incidence rate of 2227 per 100,000 person-years [95% CI: 1591, 3037]. Of the 37 cases, 29 and 8 patients with an IMID and haematologic malignancy, respectively, developed TB disease. The total follow-up time was 1084 and 558 person-years for the IMID and haematologic malignancy indication groups, respectively. Thus, the incidence rate per 100,000 person-years was higher in the IMID group, 2676 [95% CI: 1826, 3793], compared to the haematologic malignancy group, 1434 [95% CI: 666, 2723]. Although the incidence rate ratio was 1.87 [95% CI: 0.83, 4.72], it was not statistically significant (*p* = 0.06).

**Fig. 1:**
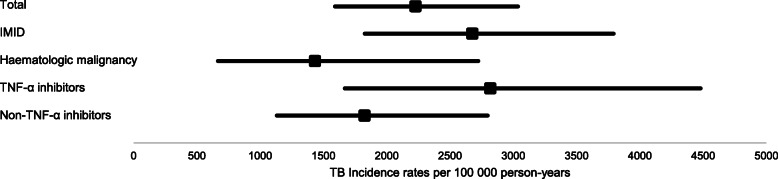
Incidence rates with 95% CI of tuberculosis disease per biologic therapies, indication and therapy groups*.* CI = confidence interval; IMID = immune mediated inflammatory disease; TNF-α = tumour necrosis factor-alpha

The overall, indication and therapy group TB incidence rates are illustrated in Fig. 1**.** Of the 37 TB disease patient cases, 16 and 19 occurred in patients who were exclusively exposed to TNF-α inhibitors and non-TNF-α inhibitors, respectively. Two patients were exposed to a combination of TNF-α and non-TNF-α biologic therapies and were therefore excluded from the therapy groups’ incidence rate calculation. The total follow-up time was 568 and 1041 person-years for the TNF-α and non-TNF-α inhibitor groups, respectively. The incidence rate of TB was higher in the TNF-α inhibitor group, 2819 [95% CI: 1669, 4480], compared to non-TNF-α inhibitor group, 1825 [95% CI: 1131, 2797]. Although the incidence rate ratio was 1.54 [95% CI: 0.74, 3.17], it was not statistically significant (*p* = 0.10).

**Fig. 2:**
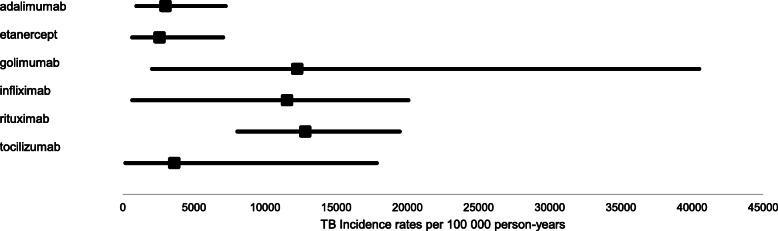
Incidence rates with 95% confidence intervals of tuberculosis disease per individual biologic therapies

The TB incidence rates per biologic therapy are illustrated in Fig. 2. The TB disease incidence rates per individual biologic therapy were calculated based on the most recent biologic therapy used before or at the time of TB disease episode (*n* = 41) divided by the sum of total cohort person-years for each biologic therapy. Incidence rates per 100,000 person-years were highest for rituximab, 12,830 [95% CI: 8055, 19,460] followed by golimumab, 12,260 [95% CI: 2056, 40,510] and infliximab, 11,550 [95% CI: 6072, 20,070]. Lower rates of 3619 [95% CI: 181, 17,850], 2997 [95% CI: 952, 7230] and 2590 [95% CI: 659, 7049] were estimated for tocilizumab, adalimumab and etanercept, respectively. Although the TB disease incidence rate was highest for rituximab, it was only significantly higher when compared to adalimumab (*p* = 0.003) and etanercept (*p* = 0.004), not when compared to the other biologic therapies. There were no TB episodes during or subsequent to abatacept or ustekinumab treatment in this study population.

The overall median time to development of TB disease was 8.3 months. (Table 2) The median time to TB was 19 and 7.4 months for patients exposed to TNF-α and non-TNF-α inhibitors, respectively. The lowest median time to TB was in patients most recently exposed to golimumab (4.8 months), followed by rituximab (7.1 months) and adalimumab (12.6 months). Estimated median times to TB disease were greater in etanercept (33.8 months), infliximab (27.7 months) and tocilizumab (15.0 months).

**Table 2:** Number of tuberculosis episodes and time to tuberculosis disease, by individual biologic therapy

Biologic therapies		Total no of patients treated^a^	No of TB episodes ^b^ (% of TB episodes/ total treated) ^c^	Time to TB (months) ^d^ Median (IQR)
All biologics		–	41	8.30 (3.67–27.70)
TNF-α inhibitors		209	20	18.95 (5.08–32.79)
	adalimumab	48	4 (8.3%)	12.56 (3.20–18.90)
	etanercept	49	3 (6.1%)	33.84 (12.59–46.82)
	golimumab	12	2 (16.7%)	4.82 (4.33–5.31)
	infliximab	100	11 (11.0%)	27.70 (3.64–39.02)
Non-TNF-α inhibitors		441	21	7.43 (2.83–16.24)
	abatacept	4	0	–
	rituximab	418	20 (4.8%)	7.08 (2.48–18.02)
	tocilizumab	18	1 (5.6%)	15.02 (15.02–15.02)
	ustekinumab	1	0	–

^a^total number of patients exposed to a biologic therapy at any point in study period (one patient may be exposed to multiple biologic therapies)

^b^number of tuberculosis episodes (single or multiple) per patient according to the most recent biologic therapy used before or at the time of tuberculosis disease diagnosis (n = 41).

^c^percentage of tuberculosis disease episodes (based on most recent biologic therapy used) per total number of patients exposed to a biologic therapy.

^d^time to tuberculosis disease in months from the start of the most recent or current biologic used to the date of tuberculosis disease diagnosis.

*TB* tuberculosis, *TNF-α* tumour necrosis factor-alpha, *min* minimum, *max* maximum, *IQR* interquartile range

A Cox proportional hazard model assessed the impact of several baseline characteristics on the time to TB incidence, as shown in Table 3. Of the variables included in the model, only a positive HIV status at biologic treatment initiation significantly contributed to development of TB (*p* = 0.01).

**Table 3:** Cox proportional hazard model for variables associated with TB disease

	*p-value*	Hazard ratio [95% CI]
Age in years	*0.35*	1.009 [0.990–1.029]
Gender *(female compared to male)*	*0.48*	1.283 [0.641–2.570]
HIV status *(HIV+ compared to HIV-)*	*0.01*	3.000 [1.262–7.127]
IPT *(IPT compared to no IPT)*	*0.32*	1.524 [0.665–3.493]
Indication groups *(IMID compared to HM)*	*0.42*	1.568 [0.523–4.698]
Therapy groups *(TNF-α inhibitors compared to non-TNF-α inhibitors)*	*0.62*	1.233 [0.543–2.801]

*IPT* isoniazid preventative therapy, *IMID* immune mediated inflammatory disease, *HM* haematologic malignancy, *TNF-α* tumour necrosis factor-alpha

## Discussion

We estimated the incidence of TB disease in public health sector patients exposed to biologic therapies in South Africa, Western Cape. We found that the estimated incidence rate among biologic therapy users was higher compared to previously published literature [22, 24]. When comparing estimated tuberculosis disease incidence rates to the estimated background incidence rate of 681 cases per 100,000 per year in the Western Cape [25], the estimated risk of tuberculosis disease is 3.3 fold higher overall, and is 4.1-fold and 2.7-fold higher than background incidence rates in TNF-α and non-TNF-α biologic therapies respectively.

Furthermore, our findings show higher incidence rates than previous local and international biologic registry findings. Our estimated TB disease incidence in patients exposed to biologic therapies (2227 per 100,000 person-years) was 1.8-fold higher than the South African Biologics Registry (SABIO) incidence rate (1240 per 100,000 person-years) [24]. This difference could be explained by both different geographical regions and socio-economic circumstances, where only the Western Cape public health sector was included in this study and majority private health sector patients throughout South Africa in the SABIO registry. International registry data, including British (BSRBR), French (RATIO) and Spanish (BIOBADASER), focused primarily on TNF-α inhibitors where estimated incidence rates varied from 106 to 172 per 100,000 person-years [22, 24]. Our estimated TB disease incidence rate among patients exposed to TNF-α inhibitors (2819 per 100,000 person-years) was therefore 16 to 27-fold higher. We hypothesise that our finding of a higher TB disease incidence rate may be a consequence of higher background TB disease risk. We found that the TB disease incidence rate ratio is 1.54 when comparing TNF-α to non-TNF-α inhibitors, which was in keeping with the findings of others [9, 10, 18].

Interestingly, the highest TB disease incidence rate for an individual biologic therapy was estimated in rituximab, a B-cell depleting agent, although its incidence rate was only significantly higher when compared to adalimumab and etanercept. Due to the mechanism of action, rituximab has been assumed to be the biologic with the lowest TB risk, and this has previously been supported by robust evidence [8, 9, 19]. Rituximab was the most commonly used biologic in this study (418 of 609 patients had rituximab exposure). A likely explanation for the high rituximab-associated TB incidence rate is the different patient profile, including patients with haematologic malignancy which poses an intrinsic risk for developing TB disease due to concomitant chemotherapy and inherent immune compromise [29]. Furthermore, we found that patients with haematologic malignancy do not routinely receive latent TB screening and isoniazid preventative therapy (IPT) in the Western Cape. In our study the overwhelming majority (98%) of patients with a haematologic malignancy did not receive IPT (illustrated in Table 1). Only 20% of patients exposed to a non-TNF-α inhibitor (of which rituximab was most frequently used) received IPT which could contribute to the high TB incidence rate estimated in rituximab exposed patients. A positive HIV status is a further confounder as four of the 19 (21%) TB positive patients who were exposed to rituximab were also HIV positive. Our findings of estimated median time to TB disease was not in keeping with previously published literature, where infliximab typically has the shortest time to TB event [20, 30, 31].

Our study has several limitations. Firstly, the PHDC collects routine clinical data to inform clinical care. It is not primarily a research data source and not all TB-related diagnostic and treatment parameters are captured digitally for individual patients. For example, the date of first TB disease evidence was inferred from the treatment start date rather than onset of symptoms, which may indicate that a diagnosis was made clinically but not captured electronically. Thus, time to development of TB may be prolonged and variable. Causes of death data were also not recorded in the PHDC. Secondly, this study has a smaller sample size (*n* = 609) compared to other studies and biologic registries [22, 24]. Moreover, four or less TB disease episodes occurred following the use of adalimumab, etanercept, golimumab and tocilizumab, respectively, which limits the ability to calculate TB disease incidence rates for individual biologic therapies and median time to TB diagnosis. Survival analysis could not be done by individual biologic therapy as patients frequently changed between and were exposed to multiple biologic therapies. Thirdly, our findings were compared to the background TB incidence where the risk was estimated per 100,000 population in the general population [25], whereas we estimated incidence rates in the population exposed to biologic therapies. In addition, when comparing our findings to other geographical areas we could only compare incidence rates in exposed patients, without considering the baseline TB risk as data on incidence rates in the general population were not available. These crude comparisons with local and international findings should therefore not be overinterpreted. Lastly, we could not accurately assess all confounders for TB risk such as which patients were assessed for IPT and the exact underlying biologic therapy indication. Specific diagnoses, such as indication for biologic therapy, was not available due to limited recording of International Classification of Diseases (ICD) codes in electronic health data. Future research should account for all TB disease risk factors and establish the indication for biologic therapy, and should be undertaken when the number of patients receiving biologic therapy has increased substantially. Ideally a more robust study design analysing prospective data should be employed to estimate causal relationships.

## Conclusion

We found higher TB incidence rates in Western Cape public sector patients exposed to both TNF-α and non-TNF-α inhibitor biologic therapies compared to the background risk. The overall TB incidence rate found in patients receiving biologic therapy in this study are generally substantially higher (estimated at approximately 3.3 fold) than incidence rates that have been reported for the Western Cape previously [25]. Due to their established efficacy, biologic therapies will become more accessible and affordable for patients accessing public health services in South Africa. It is therefore imperative to evaluate the safety profile of biologic therapies in this setting, particularly their impact on the risk of active TB infection. Latent TB screening and preventive treatment among patients exposed to biologic therapies should thus be emphasized in treatment protocols.

## Data Availability

Clinical data are available in principle from the authors upon reasonable request but restrictions may apply and permission needs to be sought from the Provincial Health Data Centre. The data source is routine clinical data and not data collected for the primary purpose of research. The data were used under license and agreement with the Provincial Health Data Centre for the current study.

## Abbreviations

BIOBADASER: Spanish Registry for Adverse Events of Biological Therapy in Rheumatic Diseases
BSRBR: British Society for Rheumatology Biologics Register
CI: Confidence interval
DMARDS: Disease modifying anti-rheumatic drugs
ETR: Electronic tuberculosis register
HIV: Human immunodeficiency virus
HM: Haematologic malignancy
ICD: International Classification of Diseases
IMID: Immune mediated inflammatory disease
IPT: Isoniazid preventative therapy
Non-TNF-α: Non-tumour necrosis factor alpha
PHDC: Provincial Health Data Centre
RATIO: Research Axed on Tolerance of Biotherapies
SABIO: South African Biologics Registry
SPSS: Statistical Product and Service Solutions
TB: Tuberculosis
TNF-α: Tumour necrosis factor alpha
WHO: World Health Organisation
